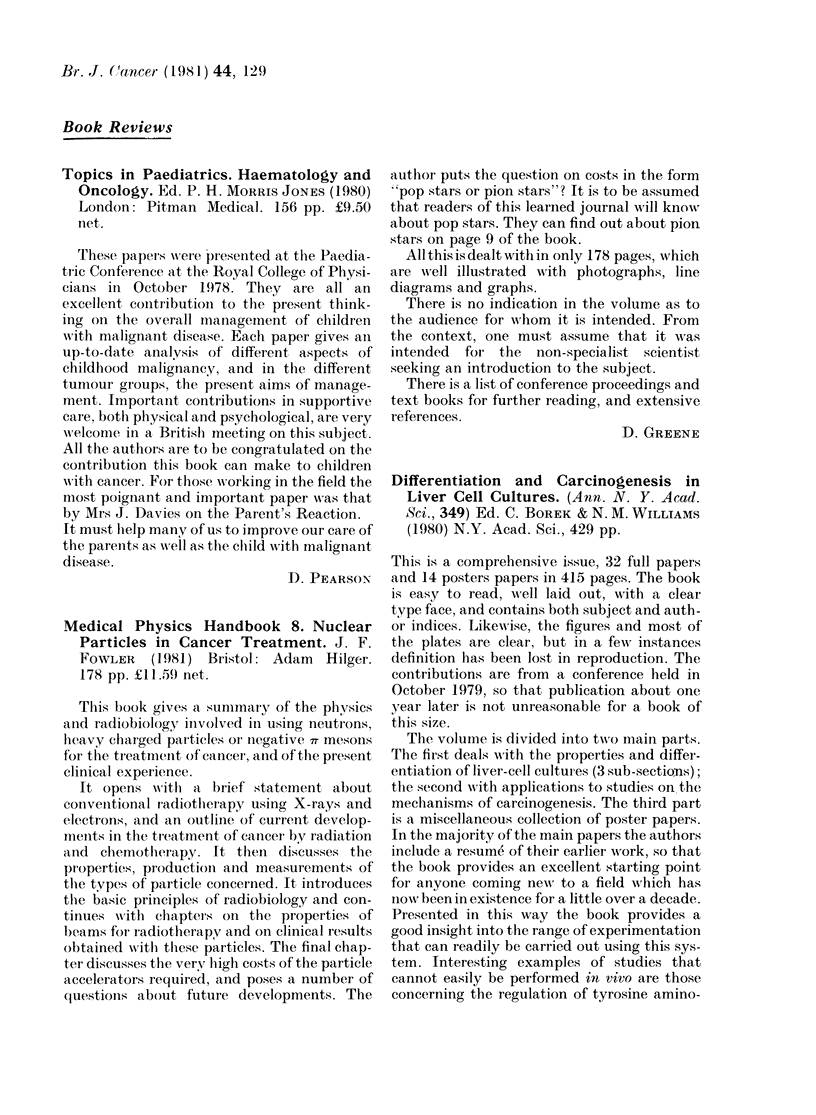# Medical Physics Handbook 8. Nuclear Particles in Cancer Treatment

**Published:** 1981-07

**Authors:** D. Greene


**Medical Physics Handbook 8. Nuclear Particles in Cancer Treatment.** J. F. Fowler (1981) Bristol: Adam Hilger. 178 pp. £11.59 net.

This book gives a summary of the physics and radiobiology involved in using neutrons, heavy charged particles or negative $\pi$ mesons for the treatment of cancer, and of the present clinical experience.

It opens with a brief statement about conventional radiotherapy using X-rays and electrons, and an outline of current developments in the treatment of cancer by radiation and chemotherapy. It then discusses the properties, production and measurements of the types of particle concerned. It introduces the basic principles of radiobiology and continues with chapters on the properties of beams for radiotherapy and on clinical results obtained with these particles. The final chapter discusses the very high costs of the particle accelerators required, and poses a number of questions about future developments. The author puts the question on costs in the form "pop stars or pion stars"? It is to be assumed that readers of this learned journal will know about pop stars. They can find out about pion stars on page 9 of the book.

All this is dealt with in only 178 pages, which are well illustrated with photographs, line diagrams and graphs.

There is no indication in the volume as to the audience for whom it is intended. From the context, one must assume that it was intended for the non-specialist scientist seeking an introduction to the subject.

There is a list of conference proceedings and text books for further reading, and extensive references.

D. Greene